# Multi-channel and free-spectral-range-free tunable add-drop filters on thin-film lithium niobate

**DOI:** 10.1515/nanoph-2025-0466

**Published:** 2025-10-28

**Authors:** Ziliang Ruan, Xijie Wang, Bin Chen, Gengxin Chen, Liu Liu

**Affiliations:** State Key Laboratory of Extreme Photonics and Instrumentation, College of Optical Science and Engineering, International Research Center for Advanced Photonics, 12377 Zhejiang University, Hangzhou, 310058, China; Jiaxing Key Laboratory of Photonic Sensing & Intelligent Imaging, Intelligent Optics & Photonics Research Center, Jiaxing Research Institute, Zhejiang University, Jiaxing, 314000, China

**Keywords:** thin-film lithium niobate, Fabry-Pérot resonator, add-drop filters

## Abstract

Thin-film lithium niobate (TFLN) has emerged as an attractive platform for integrated tunable photonic filters owing to its strong electro-optic response and low optical loss. However, conventional resonant filters, such as micro-rings, are intrinsically constrained by a limited free spectral range (FSR), which hinders their use in broadband and multi-channel operations. Here we present a TFLN-based add-drop filter that overcomes this limitation by employing a side-coupled travelling-wave Fabry–Pérot (FP) cavity formed with asymmetric multimode waveguide gratings (AMWGs). By engineering the inter-modal coupling through waveguide width tailoring and the reflection bandwidth of AMWGs, we realize a cavity with an intrinsic quality factor of 2.5 × 10^5^. The add-drop filter device also exhibits an FSR-free response across 1,500–1,630 nm wavelength band. A single resonance with a through-port extinction ratio of 20.23 dB and a drop-port insertion loss of 1.81 dB. Wavelength tuning by thermo-optic and electro-optic effect is demonstrated with efficiencies of 34.82 pm/K and 6.9 pm/V, respectively. Furthermore, a four-channel add-drop filter array with 3.2 nm channel spacing and 1.35 dB total through-port insertion loss validates the scalability of the present device. This work demonstrates an efficient approach to overcome the FSR constraint of sharp wavelength filters on TFLN. It can be potentially adopted in dense-wavelength-division-multiplexing communication systems, narrow-bandwidth microwave photonic filters, or high-resolution spectrometers.

## 1 Introduction

Driven by the increasing demands for high-performance integrated photonics, thin-film lithium niobate (TFLN) has emerged as a promising platform that uniquely combines ultra-low optical loss, large electro-optic and nonlinear coefficients, and strong mode confinement, thereby enabling compact and energy-efficient devices operating over a broad transparency window [1], [2], [3], [4]. A high-performance and flexible optical wavelength filter is one key and inevitable component on any photonic integration platforms, which has applications in optical communication, microwave photonics, quantum key distribution, and optical sensing [5], [6], [7], [8]. Wavelength filters on TFLN have also been demonstrated recently using waveguide couplers [9], Mach–Zehnder interferometers [10], Bragg gratings [11], [12], arrayed waveguide gratings [13], [14], [15], [16], [17], or optical resonators [18]. Among all these structures and technologies, the last one, i.e., optical resonator, is unique if one would like to realize wavelength filtering of an ultra-fine resolution. The most common approach to realize an optical resonator on a chip is to use a micro-ring or micro-disk cavity [19], [20]. With a good fabrication control, the quality factor of a micro-ring resonator on TFLN has now reached 10^8^, which corresponds to a propagation loss as low as 3.4 dB/m for the TFLN waveguide constructing the ring [21]. However, one drawback of a ring resonator is its finite free spectral range (FSR). This periodic resonant spectrum of a ring would introduce channel crosstalk, and would also limit the available bandwidth that can be processed separately in multi-wavelength systems [22]. Shrinking the size of a ring could somewhat relieve this problem. For example, on silicon photonics, a ring resonator of a sub-micro radius has facilitated to achieve an FSR of 93 nm covering nearly the whole C+L band [23]. Yet, on x-cut TFLN, the size of a ring is limited by the medium refractive index of the lithium niobate material, as well as the in-plane optical anisotropy [24]. So far, the FSR of a ring resonator on x-cut TFLN is still in the range of ∼10 nm [25], an order of magnitude smaller than its counterpart on silicon.

Another approach to build a resonator on a chip is to use a Fabry–Pérot (FP) resonator with waveguide Bragg gratings as reflectors [26], [27]. The cavity length of an FP resonator can be as short as only half a wavelength [28]. However, the bi-directional propagation of the optical mode inside an ordinary FP cavity would make the input and output optical beams to such a component share the same path in either end- or side-coupled configurations [29], [30]. A non-reciprocal optical device, e.g., an optical circulator, is then needed to separate them, which is difficult to realize on a chip [31]. To cope with this difficulty, asymmetric multimode waveguide gratings (AMWGs) have been proposed, which ensures the input and reflected beams from a waveguide grating in two different modes [32]. The input and output beams can then be efficiently separated using a mode multiplexer. Based on this device, high-performance optical modulators and wavelength filters have been demonstrated on TFLN [33], [34]. Yet, the commonly adopted end-coupled configuration to such a cavity still suffered from a limited available wavelength range for cascading in order to achieve multi-channel operation. Such a wavelength range is determined by the reflection spectrum of an AMWG, which is typically less than ∼20 nm on TFLN [35].

In this work, we introduce an FSR-free filter on the TFLN platform. A side-coupled FP cavity with AMWGs is adopted as the core element, which in principle enables cascading of an arbitrary number of such structures over a bus waveguide. Such a configuration has been shown to build efficient FSR-free filters on silicon [36], [37]. The proposed device exhibits a single resonance peak over a broad operating wavelength range, together with a high extinction ratio and a high quality-factor. Both all-pass filters and add-drop filters are demonstrated experimentally. Measurements further reveal an excellent thermo-optical and electro-optical tuning capability of the present device. Finally, we validate the feasibility of a four-channel add-drop filter of a high channel-spacing uniformity by cascading FP cavities of slightly different structural parameters.

## 2 Simulation and design

**Figure 1:**
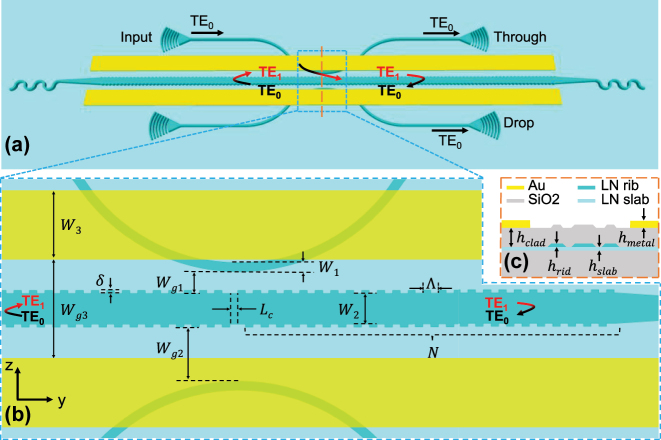
Structure of the proposed FSR-free filter on TFLN. (a) Schematic diagram of the whole device. (b) Zoom-in view of the FP cavity region showing the quarter-wave shifted region and the input/output waveguides. (c) Cross-sectional diagram of the TFLN ridge waveguide at the central position of the cavity.

Figure 1 illustrates the schematic structure of the proposed device, which consists of a FP cavity side-coupled to two input and output waveguides. The FP cavity consists of a pair of identical AMWGs serving as reflectors and a quarter-wave shifted central waveguide. The operating principle of this device can be described as follows. The fundamental transverse electric (TE_0_) mode light is injected from the input single-mode waveguide. Under phase-matching conditions, this TE_0_ mode would be coupled and converted into a forward-propagating (from left to right) first higher-order (TE_1_) mode within the wide central waveguide. When the wavelength satisfies the Bragg condition, the AMWG reflector at the right end of the FP cavity would reflect the TE_1_ mode into the TE_0_ mode in the reverse direction (from right to left). In this direction, the light would be kept in the central waveguide without out-coupling due to the large mode index mismatching to the TE_0_ mode of input single-mode waveguide. Similarly, the AMWG at the left end of the cavity would reflect the light back into TE_1_ mode, and when it propagates to the left, the mode will be partially coupled out. Another waveguide could also be brought into coupling with the central waveguide as the drop waveguide. Although this structure is physically a FP cavity, the working principle actually mimics a travelling-wave ring cavity in an add-drop configuration. It then combines the advantages of both structures, i.e., full power dropping in the output drop waveguide and an ultra-short cavity length, which help achieve an FSR-free add-drop wavelength filtering operation in the proposed device.

**Figure 2:**
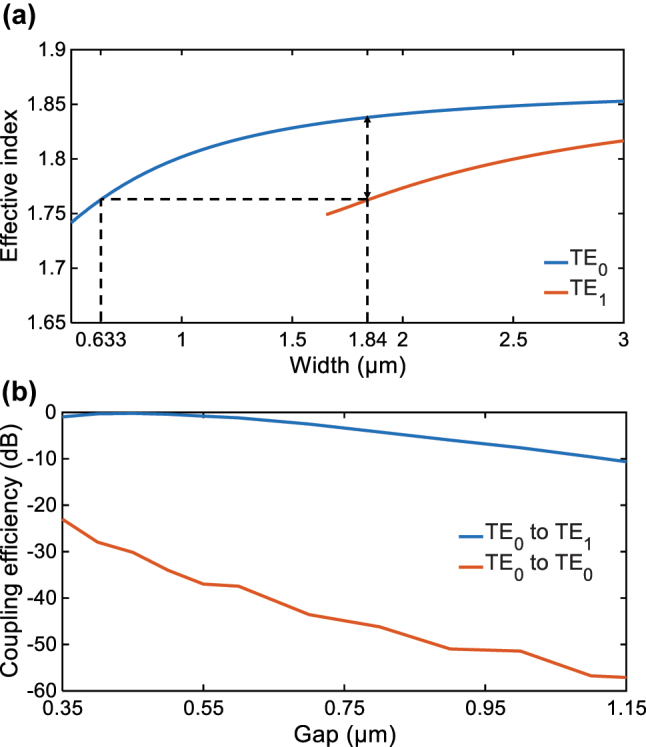
Mode coupling between the input/output waveguide and the central waveguide. (a) Relationship between the waveguide width and the effective refractive index of the TE_0_ and TE_1_ modes of the TFLN waveguide. Here, a shallowly etched TFLN waveguide with *h*_rid_ = *h*_slab_ = 200 nm is adopted. (b) Relationship between waveguide gap *w*_*g*1_ and the power coupling coefficient between the FP cavity waveguide and the through waveguide. Here, *w*_1_ = 0.633 μm and *w*_2_ = 1.84 μm is adopted. 1,550 nm wavelength is considered.

Since inter-modal coupling between distinct waveguide modes must satisfy the phase-matching condition, a key consideration of the present structure arises in choosing the width of the waveguides. To achieve selective TE_0_–TE_1_ mode coupling between the side-coupled waveguides and the FP cavity while minimizing transmission loss, we first analyzed the mode effective index properties with respect to the waveguide width, and the results are shown in Figure 2(a). In order to support two waveguide modes, the width of the FP cavity waveguide is chosen as *w*_2_ = 1.84 μm. Then the width of the through/drop waveguide is chosen as *w*_1_ = 0.633 μm to achieve the effective index matching between its TE_0_ mode and the TE_1_ mode of the FP cavity waveguide. At these widths, the effective refractive index difference between the TE_0_ modes in the two waveguides exceeds 0.08 as show in Figure 2(a), which is sufficient to avoid coupling between them. To quantitively analyze the coupling between the cavity waveguide and the through waveguide, a numerical model was constructed. The inter-modal coupling with different separation gaps *W*_*g*1_ is simulated and illustrated in Figure 2(b). Clearly, the TE_0_–TE_1_ coupling coefficient decreases rapidly with an increasing gap, consistent with the exponential decay of the modal field in the cladding layer [31]. Notably, the TE_0_–TE_0_ coupling coefficient between the two waveguides remained negligible across all tested gaps. This confirms that the selective inter-modal coupling has been effectively realized, and only the TE_1_ mode propagating from left to right in the FP cavity can interact with the side-coupled waveguides. It is also worthwhile to note that the aforementioned coupling would happen not only in the straight waveguide region in the center of the cavity, but also in the grating region. As the grating strength is weak in the present work (see below), the effective indices of the optical modes in the grating region would be almost the same as those in the central straight waveguide, which would also fulfill the index matching condition discussed above.

**Figure 3:**
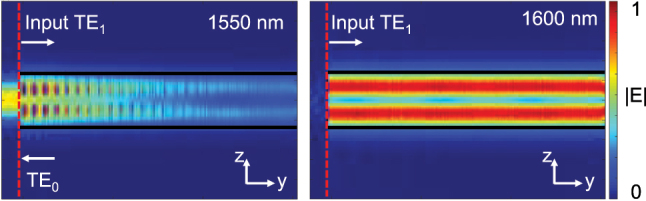
Simulated light propagation in the designed AMWGs at wavelengths of 1,550 nm and 1,600 nm.

For realizing the FSR-free single-resonance filtering, the bandwidth of the AMWG reflector should be smaller than the FSR of the FP cavity. In this design, the smallest FP cavity of only half a wavelength is adopted, which corresponding to an extra length of a quarter-wave shift *L*_c_ in the cavity. The reflectivity and bandwidth of the AMWG reflector are primarily determined by the number of grating periods (*N*) and the grating strength (*δ*). To achieve an ultra-narrow reflection bandwidth, *δ* was set to 0.06 μm. Theoretically, an AMWG reflector with *N* = 2000 yields a reflectivity of approximately 0.98. Since the operating wavelength of the device is designed around 1,550 nm, the period *Λ* of the AMWG reflector was chosen as 0.43 μm with a duty cycle of 0.5. As shown in Figure 3, at the resonant wavelength, the AMWG reflector enables efficient TE_1_-TE_0_ (and TE_0_-TE_1_) mode conversion, while the reflection of TE_0_-TE_0_ (and TE_1_-TE_1_) modes remains negligible. Outside the reflection bandwidth range, mode conversion is essentially absent.

To evaluate the tunability of the proposed add-drop filter device, two identical metal electrodes were symmetrically placed on both sides of the FP cavity. Each electrode has a width of *W*_3_ = 20 μm. To facilitate subsequent metal lift-off while minimizing the impact on the cavity mode and coupling, a relatively large electrode spacing of *W*_*g*3_ = 5 μm was set, and the thickness of the overcladding SiO_2_ layer was *h*_clad_ = 900 nm.

## 3 Fabrication and measurement

**Figure 4:**
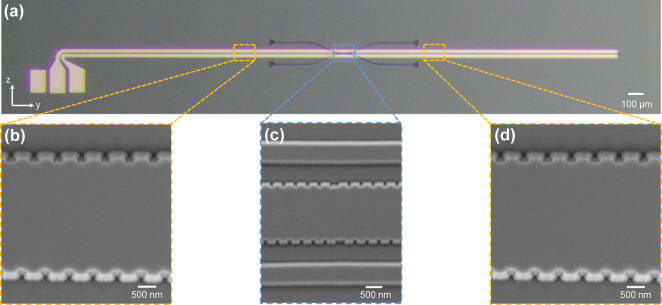
Images of the fabricated device. (a) Optical microscope image of the whole device. Scan electron microscopy images of (b) left AMWG region, (c) FP cavity region, and (d) right AMWG region.

The proposed add-drop filter chip was fabricated on a commercial lithium-niobate-on-insulator wafer. Device patterns were defined using electron beam lithography (EBL, Raith EPBG5150) and subsequently transferred onto the TFLN layer of an initial thickness of 400 nm, and the exposed area were etched down to 200 nm through argon-based inductively coupled plasma etching. A 900 nm thick SiO_2_ cladding layer was then deposited via plasma-enhanced chemical vapor deposition. Thereafter, the metal electrode patterns were formed by contact ultraviolet lithography, followed by deposition of a 5 nm titanium adhesion layer and a 300 nm gold layer using electron beam evaporation. Figure 4 shows some images of the whole fabricated filter as well as some critical regions. A broadband tunable laser system (Santec TSL-550) was used to characterize the device, and the measured spectral responses were normalized using a reference straight waveguide with grating couplers on the same chip.

**Figure 5:**
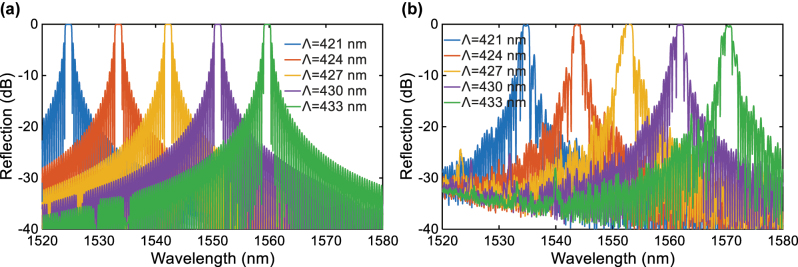
Reflection spectra of AMWGs. (a) Simulated and (b) measured reflection spectra with different periods.

As a key constituent of the FP cavity, the performance and uniformity of the AMWG directly impacts the mirror loss of the FP cavity. Poor reflectivity uniformity will increase parasitic losses and degrade the cavity’s quality factor. Meanwhile, dimensional variations in, e.g., grating period and waveguide linewidth, can induce cavity mode shifts, thereby deteriorating the accuracy of the filter’s center wavelength. To validate the AMWG’s performance, Figure 5(a) and (b) present the simulated and measured reflection spectra of AMWGs with five different grating periods (spaced by 3 nm). Although there are still discrepancies in the absolute central wavelengths of the reflection spectra, the amount of wavelength shifts with respect to variations in grating periods reveal excellent agreement between simulation and experimental results. This is particularly critical for the present FP cavity filter, as it guarantees consistent channel spacing when scaling to multi-channel arrays. Presently, further increasing the grating length would not help improve the reflectivity of the AMWG, but introduce more side-lobe peaks in the spectrum. This is mainly due to the uniformity of the grating period from the fabrication.

**Figure 6:**
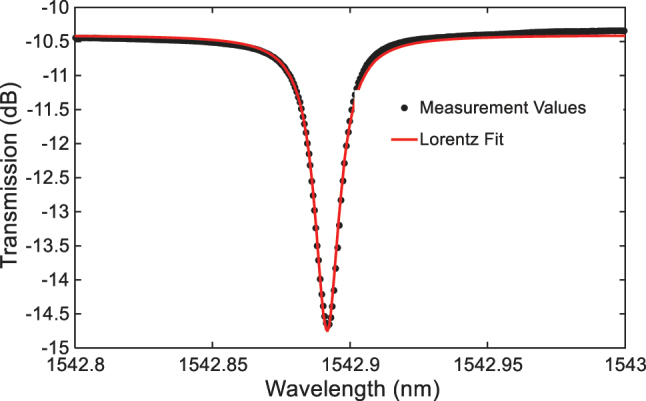
The measured spectral response of the fabricated all-pass FP cavity and its Lorentz fit.

To quantify the performance of the FP cavity filter formed by AMWG reflectors, we first characterized its quality factor. As a key metric reflecting the cavity loss and resonance sharpness, the quality factor directly determines the device’s narrowband filtering capability. Here, an all-pass configuration is adopted, where the drop waveguide is absent, or *W*_*g*2_ = ∞. By optimizing the grating waveguide gap *W*_*g*1_ to operate the FP cavity near critical coupling, the normalized spectral response at the FP cavity at the through port is shown in Figure 6 together with a fit to the Lorentz line shape. The extracted intrinsic quality factor reached 2.5 × 10^5^, confirming an efficient light confinement within the cavity with a low loss. Using apodization at the edge of the AMWG would help further improving the quality factor of the present cavity.

**Figure 7:**
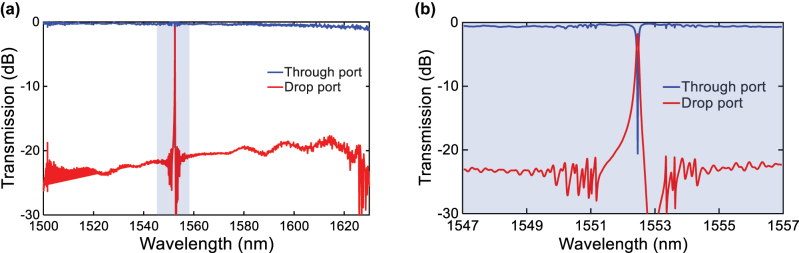
Measured normalized spectral responses of the fabricated add-drop filter. (a) Spectral reponses at the through and drop ports in 130 nm wavelength range. (b) Zoom-in view near the resonant wavelength.

Subsequently, the tunable add-drop filter as shown in Figure 4(a) was tested. The fabricated device presents a symmetrical input and output coupling to the cavity, i.e., *W*_*g*1_ = *W*_*g*2_ = 500 nm. Figure 7(a) show the measured normalized spectral responses of one fabricated device at the through port and drop port across a wide wavelength range covering 1,500–1630 nm, which is limited by the tunable laser. Clearly, only a single resonance peak exists. Figure 7(b) shows a zoom-in view near the resonance. As expected, the resonance has shifted from the nominal 1,550 nm–1,552.46 nm. Nonetheless, at resonance, the through port exhibits an extinction ratio of 20.23 dB and a 3-dB bandwidth of 0.7 nm. At the drop port, an insertion loss of 1.81 dB can be obtained. These results verify that the proposed side-coupled FP cavity with AMWGs indeed help achieve FSR-free operation over the measured 130 nm wavelength range. It should be noted that, as shown in Figure 7(b), an asymmetric spectrum at the drop port can be observed, which results from the Fano resonance phenomenon caused by the interference of the narrowband resonant signal from the FP cavity and the broadband non-resonant background signal from the direct coupling between the input and drop waveguides. Increasing the spacing between the coupling gaps (*W*_*g*1_ and *W*_*g*2_) can reduce this asymmetry for the drop port spectrum.

**Figure 8:**
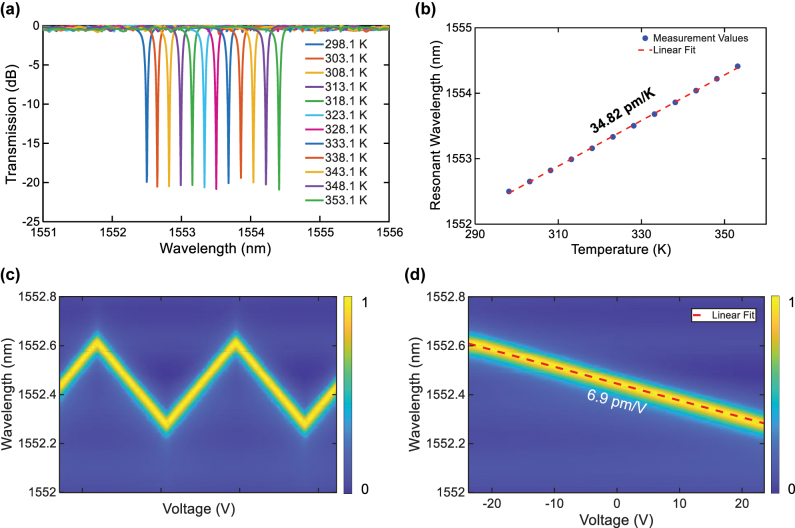
Tuning properties of the faricated filter. (a) Normalized transmission spectra at different chip temperatures. (b) Curve of resonant wavelengths versus temperatures. (c) Electro-optic tuning of the device. (d) Zoom-in view of a region in (c).

The tunability of the fabricated add-drop filter was further investigated through thermo-optic and electro-optic tuning. For the former case, the chip was placed on a hotplate, and the temperature was increased from 298.1 K to 353.1 K at a step of 15 K. Figure 8(a) shows the normalized transmission spectra at different temperatures. A linear fit yields a thermal sensitivity of the resonance wavelength of 34.82 pm/K as shown in Figure 8(b). For electro-optic tuning, the chip was mounted on a temperature-controlled stage. To avoid low-frequency drift, a triangular-wave driving signal with a peak-to-peak voltage of 50 V and frequency of 10 kHz was applied to the electrodes using a function generator. Figure 8(c) presents the relationships among the resonance wavelength, the through port transmission near the resonant wavelength, and the driving voltage. Linear fitting of the resonance wavelength as a function of the driving voltage reveals that the present device exhibits an electro-optic tuning efficiency of 6.9 pm/V. Due to their distinct tuning mechanisms, the two tuning methods exhibit significant differences in the response speed. They can be used in different scenarios. For example, the electro-optic tuning handles high-speed dynamic modulations, while the thermo-optic tuning manages static baseline calibrations.

**Figure 9:**
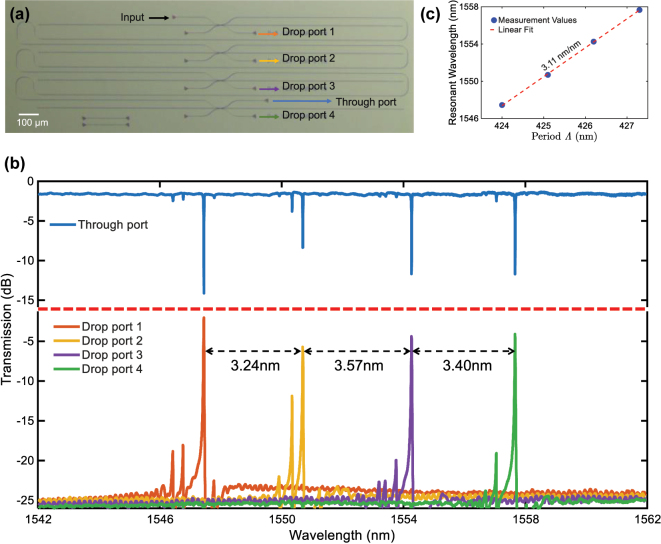
Structure and performance of the cascaded filter array. (a) Fabricated four-channel filter array by cascading four side-coupled FP cavities, (b) Measured spectral responses of the filter array. (c) Resonant wavelength of each channel and the corresponding grating period *Λ*.

Finally, to prove the feasibility of the multi-wavelength filtering capability based on the present device, a four-channel filter array, as illustrated in Figure 9(a), was designed and fabricated. This array comprises four FP cavities with AMWG reflectors, and they are cascaded on a common through waveguide. In order to achieve a target channel spacing of 3.2 nm (400 GHz) in accordance with the phase-matching condition, the grating periods *Λ* of these AMWG reflectors were set to 424.0 nm, 425.1 nm, 426.2 nm, and 427.3 nm, respectively. Figure 9(b) presents the normalized output spectrum of the array. The results indicate that the insertion loss of the device is about 1.35 dB, and the channel spacings between adjacent channels were measured to be 3.2 nm, 3.6 nm, and 3.4 nm, respectively. As shown in Figure 9(c), the filter array exhibits excellent channel uniformity consistency, which gives a structural tuning sensitivity of 3.11 nm/nm for the resonant wavelength to the grating period.

In the four-channel filter array, some side-lobe peaks can be observed in the drop port responses in Figure 9(b), which are mainly attributed to two factors: slight variations and nonuniformity in the AMWG period introduced during the fabrication. These two factors collectively shift the resonance of the FP cavity from the center of the AMWG reflection spectrum, and give some pronounced side-lobe reflection peaks of the reflection as shown in Figure 5(b). Consequently, the extinction ratio of the four-channel array is overall lower than that of the single-channel device. To alleviate this issue, strategies of improving the fabrication precision and optimizing the AMWG design by introducing apodizations in the grating boundaries can be adopted.

Theoretically, filter arrays with a larger number of channels and smaller channel spacing can be realized by adjusting the number of cascaded filters and the periods of the reflectors. Independent wavelength compensation for each channel can be achieved by equipping the FP cavity structure of each channel with a separate local heater. This facilitates to ensure a uniform channel spacing and compensate the fabrication variations.

## 4 Conclusions

In summary, we have demonstrated a multi-channel FSR-free add-drop filter on the TFLN platform. The device is based on a travelling-wave FP cavity enable by AMWG reflectors. First, inter-modal coupling has been designed by tailoring the waveguide widths to satisfy the phase-matching condition. The AMWG facilitating a narrow-band Bragg reflection has been designed and realized, whose high reflectivity and low loss helps achieve a cavity quality factor of 2.5 × 10^5^. Secondly, add-drop filters with symmetric sided-coupled waveguides on both sides of the FP cavity exhibits a single resonance peak in 1,500–1,630 nm wavelength range, confirming its FSR-free characteristic. At resonance, the device achieves an extinction ratio of 20.23 dB and a drop-port insertion loss of 1.81 dB. Tunability tests reveal a thermal sensitivity of 34.82 pm/K and an electro-optic tuning efficiency of 6.9 pm/V for the present device, enabling a flexible wavelength reconfigurability via both temperature or voltage signals. Finally, a four-channel filter array has been demonstrated, with a channel spacing of 3.2 nm and an insertion loss <1.35 dB. The performances of the proposed device, including, the high-quality factor, the FSR-free filtering, the tunability, and scalability, collectively confirm its promise in large-capacity optical data transmission (e.g., wavelength multiplexing systems), high-resolution optical spectral sensing, and microwave photonics applications.

## References

[1] Wang C. (2018). Integrated lithium niobate electro-optic modulators operating at CMOS-compatible voltages. *Nature*.

[2] Hu Y. (2025). Integrated electro-optics on thin-film lithium niobate. *Nat. Rev. Phys.*.

[3] He M. (2019). High-performance hybrid silicon and lithium niobate Mach–Zehnder modulators for 100 Gbit s^−1^ and beyond. *Nat. Photon.*.

[4] Capmany J., Novak D. (2007). Microwave photonics combines two worlds. *Nat. Photonics*.

[5] Han L. (2022). Integrated Fabry–Perot filter with wideband noise suppression for satellite-based daytime quantum key distribution. *Appl. Opt.*.

[6] Yoo K. M. (2023). Lab-on-a-chip optical biosensor platform: A micro-ring resonator integrated with a near-infrared Fourier transform spectrometer. *Opt. Lett.*.

[7] Yu S. (2023). On-chip single-mode thin-film lithium niobate Fabry–Perot resonator laser based on Sagnac loop reflectors. *Opt. Lett.*.

[8] Huang Q. (2025). On-chip tunable single-mode high-power narrow-linewidth Fabry–Perot microcavity laser on Yb^3+^-doped thin-film lithium niobate. *Photonics Res.*.

[9] Mo Z., Li C., Chen G., Zeng C., Xia J. (2025). Ultrabroadband spectral filter on lithium-niobate-on-insulator. *ACS Photonics*.

[10] Yu L. (2024). Ultra-compact and high-performance four-channel coarse wavelength-division (De)multiplexing filters based on cascaded Mach–Zehnder interferometers with bezier-shape directional couplers. *Opt. Express*.

[11] He J. (2023). First realization of a three-channel lithium-niobate photonic filter for 50G passive optical networks. *ACS Photonics*.

[12] Prencipe A., Baghban M. A., Gallo K. (2021). Tunable ultrannarrowband grating filters in thin-film lithium niobate. *ACS Photonics*.

[13] Yi J. (2024). Anisotropy-free arrayed waveguide gratings on X-cut thin film lithium niobate platform of in-plane anisotropy. *Light: Sci. Appl.*.

[14] Yu Y., Yu Z., Zhang Z., Tsang H. K., Sun X. (2022). Wavelength-division multiplexing on an etchless lithium niobate integrated platform. *ACS Photonics*.

[15] Tu H. (2024). 100-Channel arrayed waveguide grating based on thin film lithium niobate on insulator (LNOI). *J. Lightwave Technol.*.

[16] Wang Z. (2025). An electro-optically tunable arrayed waveguide grating fabricated on thin film lithium niobate. *APL Photonics*.

[17] Wang Z. (2024). On-Chip arrayed waveguide grating fabricated on thin-film lithium niobate. *Adv. Photonics Res.*.

[18] Hou S. (2023). Programmable optical filter in thin-film lithium niobate with simultaneous tunability of extinction ratio and wavelength. *ACS Photonics*.

[19] Han M., Li J., Wei C., Liu J. (2023). Ultra-wideband tunable microwave photonic filter based on thin film lithium niobate. *Photonics*.

[20] Wu R. (2018). Lithium niobate micro-disk resonators of quality factors above 10^7^. *Opt. Lett.*.

[21] Gao R. (2022). Lithium niobate microring with ultra-high Q factor above 10^8^. *Chin. Opt. Lett.*.

[22] Ouyang B., Xing Y., Bogaerts W., Caro J. (2019). Silicon ring resonators with a free spectral range robust to fabrication variations. *Opt. Express*.

[23] Liu D., Zhang C., Liang D., Dai D. (2019). Submicron-resonator-based add-drop optical filter with an ultra-large free spectral range. *Opt. Express*.

[24] Wang J., Chen P., Dai D., Liu L. (2020). Polarization coupling of X-cut thin film lithium niobate based waveguides. *IEEE Photonics J*..

[25] Liu X. (2024). Sharp Bend and large FSR ring resonator based on the free-form curves on a thin-film lithium niobate platform. *Opt. Express*.

[26] Liu Q., Zeng D., Mei C., Li H., Huang Q., Zhang X. (2022). Integrated photonic devices enabled by silicon traveling wave-like Fabry–Perot resonators. *Opt. Express*.

[27] Zhu M. (2025). Multichannel lithium-niobate-on-insulator photonic filter for dense wavelength-division multiplexing. *ACS Photonics*.

[28] Novotny L., Hecht B. (2012). *Principles of Nano-Optics*.

[29] Abdelsalam K. (2021). Tunable dual-channel ultra-narrowband bragg grating filter on thin-film lithium niobate. *Opt. Lett.*.

[30] Sun C. (2022). Tunable narrow-band single-channel add-drop integrated optical filter with ultrawide FSR. *PhotoniX*.

[31] Zhang M. (2019). Broadband electro-optic frequency comb generation in a lithium niobate microring resonator. *Nature*.

[32] Wang X. (2014). Precise control of the coupling coefficient through destructive interference in silicon waveguide bragg gratings. *Opt. Lett.*.

[33] Pan B. (2022). Compact electro-optic modulator on lithium niobate. *Photonics Res.*.

[34] Liu H. (2024). First demonstration of lithium niobate photonic chip for dense wavelength-division multiplexing transmitters. *Adv. Photonics*.

[35] Sun C. (2021). Free-spectral-range-free filters with ultrawide tunability across the S + C + L band. *Photonics Res.*.

[36] Ma Y. (2021). Silicon add-drop multiplexer based on π phase-shifted antisymmetric Bragg grating. *IEEE J. Quantum Electron.*.

[37] Yariv A. (1973). Coupled-mode theory for guided-wave optics. *IEEE J. Quantum Electron.*.

